# Correlation of structural defects in the ascending aortic wall to ultrasound parameters: benefits for decision-making process in aortic valve surgery

**DOI:** 10.1186/s13019-017-0671-8

**Published:** 2018-01-18

**Authors:** Saša D. Borović, Milica M. Labudović Borović, Ivan V. Zaletel, Vera N. Todorović, Petar A. Dabić, Jelena T. Rakočević, Jelena M. Marinković-Erić, Predrag S. Milojević

**Affiliations:** 1 Dedinje Cardiovascular Institute, Belgrade, Serbia, 1 Heroja Milana Tepića Street, Belgrade, 11000 Serbia; 2 Institute of Histology and Embryology “Aleksandar Đ. Kostić”, Faculty of Medicine, University of Belgrade, 26 Višegradska Street, Belgrade, 11000 Serbia; 3 Faculty of Stomatology, University Business Academy in Pančevo, Novi Sad, Serbia; 4 Institute of Medical Statistics, Faculty of Medicine, University of Belgrade, Belgrade, Serbia

**Keywords:** Aortic stenosis, Ascending aorta replacement, Aortic valve replacement, Aging, Remodeling

## Abstract

**Background:**

Histopathological changes in the ascending aorta wall in patients with severe tricuspid aortic valve (TAV) stenosis were graded and correlated to echocardiographic parameters. Objective was to associate threshold echocardiographic values with structural defects in the ascending aorta providing a tool to improve decision-making process in cases when simultaneous aortic valve replacement (AVR) and ascending aorta replacement is considered.

**Methods:**

Biopsies from 108 TAV stenosis patients subjected to AVR were graded into three grades according to severity of aortic wall changes. Echocardiographic parameters obtained preoperatively and correlated to grade, age, gender and risk factors, were diameters of ventriculo-aortic junction (AA), sinus Valsalva (SV), sinotubular junction (STJ), the largest diameter of the visualized ascending aorta (AscA) as well as indexes: sinus Valsalva (SVI), sinotubular junction (STJI), AscA/AA and STJ/AA.

**Results:**

Two echocardiographic parameters portrayed grades with statistical significance: STJ (F = 5.417; *p* = 0.006 (*p* < 0.05)) and AscA (F = 3.924; *p* = 0.023 (p < 0.05)). By using multiple predictors in the setting of Regression analysis, statistically significant differences among grades were reached for AA, SV, STJ, AscA and SVI. With further ROC curves analysis, threshold values for different grades were recognized. Grade 2 is identified in patients with AscA > 3.3 cm, while Grade 3 is identified in patients with values of AscA > 3.5 cm, STJ > 2.9 cm and STJI > 1.

**Conclusions:**

Hemodynamic stress induced by TAV stenosis leads to elastic lamellae disruption in the aortic wall. Those changes could be graded and correlated with echocardiographic parameters of the aortic root and ascending aorta, providing a tool for decision to replace ascending aorta concomitantly with AVR.

## Background

In the present study, we investigated the spectrum of structural changes in the ascending aortic wall in patients with severe degenerative, calcific aortic stenosis of the tricuspid aortic valve (TAV), and correlated them to echocardiographic parameters. It is important to understand the evolution of aortic wall changes due to aortic stenosis, for tailoring guidelines for surgical treatment of aortic stenosis. Current guidelines recommend that simultaneous surgery of the aortic root and ascending aorta should be considered in patients with degenerative TAV stenosis, when maximal ascending aortic diameter is ≥55 mm. The main goal of our study is to determine when is the replacement of the ascending aorta warranted simultaneously with the aortic valve replacement (AVR), from the histological perspective.

We addressed several issues in this paper. The influence of severe TAV stenosis on structural changes in the wall of ascending thoracic aorta. Design and application of the grading system that identifies gradual progression of aortic wall changes caused by hemodynamic disturbances in the setting of the aortic stenosis. Definition of irreversible changes in the ascending aorta wall in patients with severe aortic stenosis, Correlation of histological grades with echocardiographic parameters in order to obtain reliable insight into the aortic wall structure by means of non-invasive diagnostics.

The answers to issues are particularly complex because aortic stenosis and hemodynamic derangement that it causes, is not the only factor influencing the structure of the ascending aorta. Other factors including aging, arterial hypertension, atherosclerosis and diabetes mellitus may act synergistically resulting in definitive changes [1–3]. Finally, we analyzed are there any gender-related differences in the remodeling process.

We focused exclusively on the severe TAV stenosis and its influence on the ascending aorta wall. The chosen method was to compare grades of elastic skeleton defects, assessed by light microscopy, with the echocardiographic parameters. Grading of the structural changes was done according to accepted grading systems.

## Methods

### Overall patients data

We performed analysis of wall segments of the ascending aorta of 108 patients who were undergoing AVR because of severe, symptomatic TAV stenosis. All patients were operated at Dedinje Cardiovascular Institute. There were 56 (51.9%) males and 52 (48.1%) female patients. The mean age of patients was 67.56 ± 8.23 years. The mean age of male patients was 67.23 ± 8.49 years (median 68.5 (60–74)), while for the female patients it was 67.92 ± 8.01 years (median 70 (59.75–74)). There was no statistical significance in the mean age of male and female patients in the aortic stenosis group (Mann-Whitney U test: 1403.500; *p* = 0.747 (> 0.05)).

Diameter of the ascending aorta was <5 cm in all patients, with the mean value of 3.33 ± 0.54 cm. The minimal diameter was 2.2 cm and the maximal diameter 4.7 cm.

Excluded from this study group were (1) patients with moderate or severe aortic regurgitation, (2) patients with aortic stenosis and acute or chronic aortic dissection, (3) patients who had had a previous cardiac operation and (4) patients who had had aortic stenosis combined with a connective tissue disorder, bicuspid or congenitally malformed aortic valve.

### Intraoperative Aortic Wall sampling

The diagnosis of a severe TAV stenosis was established by preoperative echocardiography. Transverse aortotomy was made approximately 1 cm above the take-off of the right coronary artery, slightly above the level of the sinotubular junction. The aortic wall specimens were taken from the distal lip of the incision at the convexity of the ascending aorta, 2 to 4 cm above the level of the aortic valve annulus [4]. Samples of the aortic wall with the minimal dimensions 1 mm × 9 mm and maximal dimensions 5 mm × 20 mm were excised, immediately fixed in 4% neutral buffered formaldehyde by the immersion procedure, and subsequently processed for the morphological and morphometric analysis.

### Echocardiographic parameters

Echocardiographic parameters of the aortic root and the ascending aorta were determined preoperatively from parasternal longitudinal section with standard 2D procedure. Diameters at the level of ventriculo-aortic junction (AA), sinus Valsalva (SV), sinotubular junction (STJ) and the largest diameter of visualized ascending aorta (AscA) were measured.

Index of sinus Valsalva (SVI) was calculated as the ratio between measured and predicted diameter (pSV) at the level of the sinus of Valsalva. Predicted diameter at the level of the sinus Valsalva (pSV) was calculated according to regression formula pSV(cm) = 1,92 + 0,74xBSA(m^2^), where BSA stands for body surface area [5]. Sinotubular junction index (STJI) was calculated as the ratio between measured and predicted diameter at the level of sinotubular junction. Predicted diameter at the level of sinotubular junction was calculated according to regression formula pSTJ(cm) = 1,69 + 0,62xBSA(m^2^) [5].

Indexes AscA/AA and STJ/AA were calculated as the ratio between AscAA or STJ diameters and AA diameter, respectively.

### Preparation of arterial samples for analysis

#### Preparation of tissue for light microscopy and Histomorphometry

The tissue was prepared for morphological and morphometric analysis according to the procedure described in the previously published studies of our group [6–8]. Out of 30 serial sections per patient, three sections were chosen for the analysis with respect to following rules: oblique sections were excluded from the analysis, as well as sections with major technical flaws. In addition, minimal distance between chosen sections must be at least 100 μm.

Sections were stained with the application of selective techniques for elastic fibers: Weigert van Gieson technique with resorcin fuchsine, Verhoeff van Gieson method or Pincus’ staining with acid orcein.

#### Grading of morphological changes

Grading of morphological changes in the ascending aorta was established to test the hypothesis that aortic stenosis induces progressive histopathological changes and that subsequent grades follow the natural history of these alterations. All sections were graded according to the principle described by Schlatmann and Becker [9] for gradation of aortic wall changes during aging and Niwa et al. [10] for the gradation of congenital aortic stenosis.

Using both methods for morphometric analyses of elastic skeleton parameters, we found statistically significant differences among the grades (data not shown). However, we decided to proceed with Schlatmann and Becker gradation system since criteria for grading were more precise, hence, the reproducibility of the results was also higher with this system.

The grades were established according to the most severe changes at the magnification ×200 of the Olympus BX41 microscope.

**Fig. 1:**
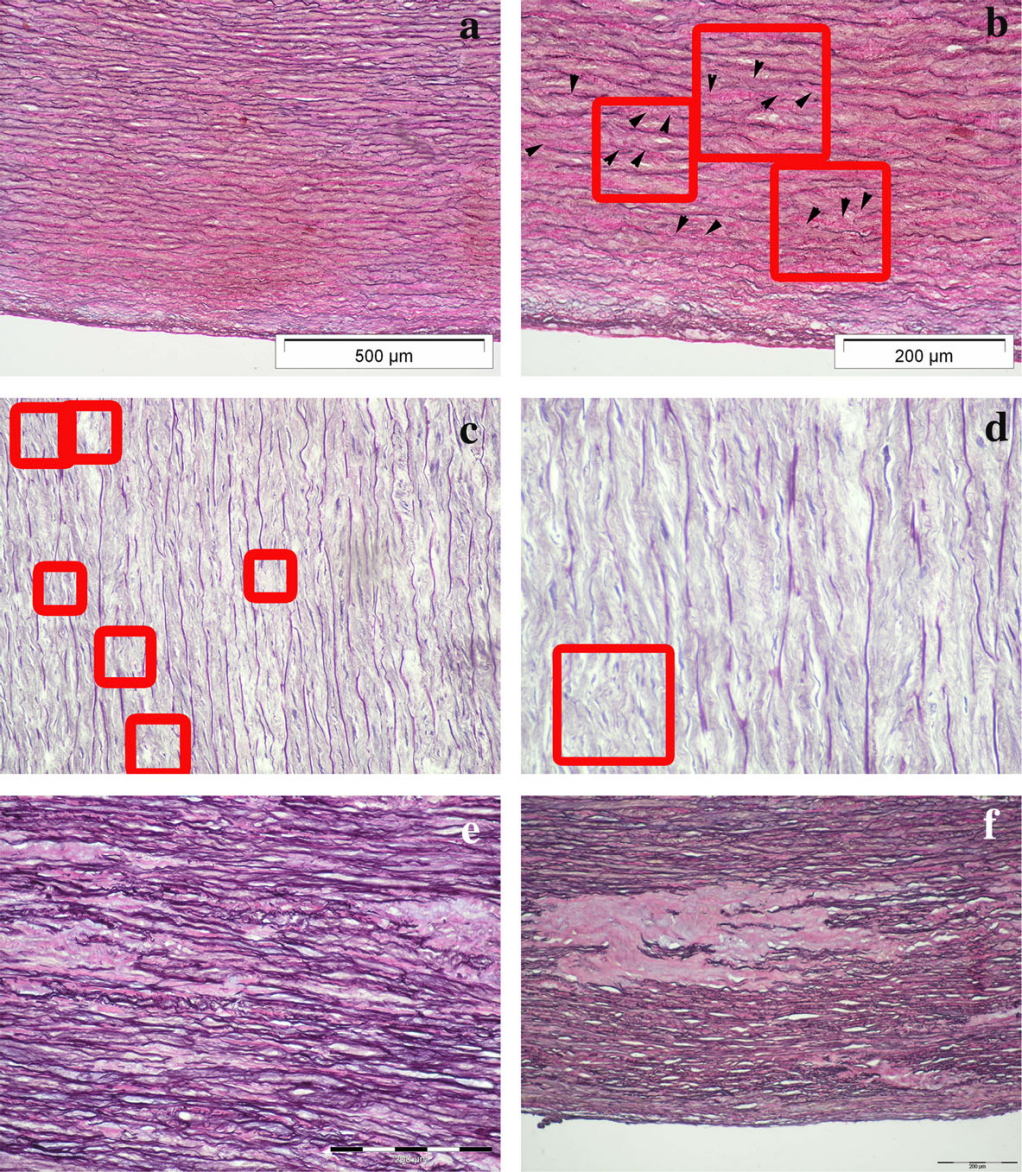
The ascending aorta in patients with severe aortic stenosis – **a**) and **e**) (Weigert van Gieson staining, original magnification ×100, bar = 500 μm); **b**) and **f**) (Weigert van Gieson staining, original magnification ×200, bar = 200 μm); **c**) (PTAH staining, original magnification ×200, bar = 200 μm); **d**) (PTAH staining, original magnification, ×400): **a) – b)** grade 1; three foci of elastic lamellae fragmentation in 1 microscopic field of the Olympus BX41 microscope, magnification ×200; focus of elastic lamellae fragmentation comprises 2 to 4 neighboring elastic lamellae; **c)** grade 2; eight foci of elastic lamellae fragmentation in 1 microscopic field of the Olympus BX41 microscope, magnification ×200; confluent or scattered foci throughout the media of the aorta; **d) - f)** the presence of foci with elastic fragmentation in 10 or more neighboring elastic lamellae. As opposite to control aortas, aortas of aortic stenosis patients have thin subendothelial connective tissue with numerous elastic fibers. These samples are atherosclerosis free or with low grade atherosclerosis (types I – III atherosclerotic lesions)

**Grade 1** slides had fewer than five foci of elastic lamellae fragmentation in one microscopic field. Focus of elastic lamellae fragmentation comprises 2 to 4 neighboring elastic lamellae (Fig. 1a-b).

**Grade 2** sections had 5 to 10 foci of elastic lamellae fragmentation in one microscopic field and foci were confluent or scattered throughout the media of the aorta (Fig. 1c-d).

**Grade 3** sections were distinguished by the presence of foci with elastic fragmentation in 10 or more neighboring elastic lamellae, irrespective of the number of foci per microscopic field, with disorganization of smooth muscle cells layers (Fig. 1e-f).

Pathologist, who performed the analysis, was blinded for patients’ data. The slides were reexamined twice to obtain the final data as advised in previous similar studies [11].

Atherosclerosis was graded according to established classification systems [12].

### Statistical analysis

Descriptive statistics included the mean values or the median with 25th – 75th percentile values, the standard deviation (SD), the standard error (SE) and a 95% confidence interval (95% C.I.).

The tests were performed with the SPSS version 10.0 for Windows. Following tests were used where appropriate: One-Sample Kolmogorov-Smirnov Test, ANOVA, Mann-Whitney test, Bonferroni Post Hoc Multiple Comparison test, Tukey Post Hoc, Pearson Correlation and Spearman’s Rho, ROC Curves and Regression Analysis. The value of *p* < 0.05 was considered statistically significant.

Data are presented as the means ± SD or the median with 25th – 75th percentile value.

#### Data distribution pattern – Echocardiographic parameters

Values of echocardiographic parameters conform normal distribution as confirmed with the One-Sample Kolmogorov-Smirnov Test, hence they were analyzed with parametric tests.

## Results

### Overall patients’ data

Overall patients’ data are systematized in Table 1.

**Table 1:** Baseline clinical characteristics

Number of patients (n)	108	
Age (years) (mean ± SD)	67.56 ± 8.23	
Males (years) (median (25th – 75th percentile))	68.5 (60–74)	
Females (years) (median (25th – 75th percentile))	70 (59.75–74)	
Parameter	Number of patients (n)	Percentage (%)
Arterial hypertension (HTA)	80	74.1
Diabetes mellitus (DM)	26	24.1
Coronary artery disease (CAD)	35	32.4
Chronic renal disease (ChRD)	18	16.7
Chronic pulmonary disease (ChPD)	11	10.2
Peripheral vascular disease (PVD)	22	20.4

The distribution of grades are presented in Table 2.

**Table 2:** Grades distribution

Grades	Number of patients (n)	Percentage (%)
Grade 1	63	58.3
Grade 2	27	25
Grade 3	18	16.7
Gender		
Males (total)	56	51.9
Grade 1	29	51.79
Grade 2	13	23.21
Grade 3	14	25
Females (total)	52	48.1
Grade 1	34	65.38
Grade 2	14	26.92
Grade 3	4	7.69
Number of patients (n)	108	

### Echocardiographic parameters and grades

**Fig. 2:**
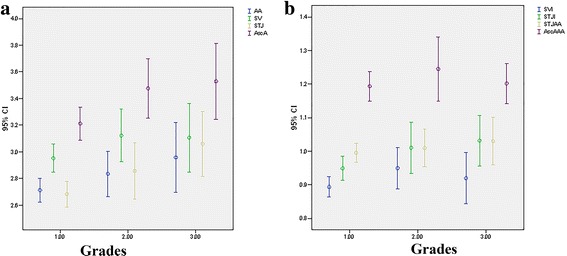
Values of echocardiographic parameters in different grades: **a**) values of diameters at the level of ventriculo-aortic junction (AA), sinus Valsalva (SV), sinotubular junction (STJ) and the largest diameter of visualized ascending aorta (AscA); **b**) values of sinus Valsalva index (SVI), sinotubular junction index (STJI), STJ/AA and AscA/AA

Values of echocardiographic parameters are given in Table 3. With the increase in grade and severity of histopathological defect, values of echocardiographic parameters increase (Fig. 2). Statistical significance was confirmed with ANOVA among echocardiographic parameters of different grades for STJ (F = 5.417; *p* = 0.006 (*p* < 0.05)) and AscA (F = 3.924; *p* = 0.023 (p < 0.05)) (Table 2 and Fig. 2). By using Bonferroni Post Hoc Analysis statistical significance among grades was confirmed for STJ, but not for AscA (GR1 vs GR2 *p* = 0.079; GR 1vs GR3 *p* = 0.093; GR2vs GR3 *p* = 1.000).

**Table 3:** Echocardiographic parameters of different morphological grades

Parameter	Grades	N	Mean	SD	SE	95% C.I.	Min.		Max.	p
AA	1.00	63	2.7122	0.35304	0.04448	2.6233	2.8011	2.00	3.50	0.062
	2.00	27	2.8344	0.42827	0.08242	2.6650	3.0039	2.20	4.20	
	3.00	18	2.9578	0.52635	0.12406	2.6960	3.2195	1.97	4.10	
	Total	108	2.7837	0.41178	0.03962	2.7052	2.8623	1.97	4.20	
SV	1.00	63	2.9519	0.42024	0.05294	2.8461	3.0577	2.00	3.90	0.190
	2.00	27	3.1219	0.49951	0.09613	2.9243	3.3195	2.10	4.40	
	3.00	18	3.1067	0.51923	0.12238	2.8485	3.3649	2.40	4.50	
	Total	108	3.0202	0.46087	0.04435	2.9323	3.1081	2.00	4.50	
STJ	1.00	63	2.6830	0.38806	0.04889	2.5853	2.7807	2.00	3.60	0.006*
	2.00	27	2.8570	0.53146	0.10228	2.6468	3.0673	2.00	3.80	
	3.00	18	3.0594	0.49402	0.11644	2.8138	3.3051	2.40	4.20	
	Total	108	2.7893	0.46341	0.04459	2.7009	2.8777	2.00	4.20	
AscA	1.00	63	3.2124	0.49394	0.06223	3.0880	3.3368	2.20	4.60	0.023*
	2.00	27	3.4767	0.56276	0.10830	3.2540	3.6993	2.60	4.70	
	3.00	18	3.5289	0.57376	0.13524	3.2436	3.8142	2.67	4.70	
	Total	108	3.3312	0.53931	0.05190	3.2283	3.4341	2.20	4.70	
SVI	1.00	63	0.8935	0.12078	0.01522	0.8631	0.9239	0.63	1.22	0.199
	2.00	27	0.9493	0.15402	0.02964	0.8883	1.0102	0.64	1.28	
	3.00	18	0.9194	0.15345	0.03617	0.8431	0.9958	0.71	1.34	
	Total	108	0.9118	0.13609	0.01309	0.8858	0.9377	0.63	1.34	
STJI	1.00	63	0.9489	0.14145	0.01782	0.9133	0.9845	0.68	1.44	0.073
	2.00	27	1.0100	0.19365	0.03727	0.9334	1.0866	0.71	1.37	
	3.00	18	1.0317	0.15143	0.03569	0.9564	1.1070	0.84	1.44	
	Total	108	0.9780	0.15997	0.01539	0.9474	1.0085	0.68	1.44	
STJ/AA	1.00	63	0.9954	0.11409	0.01437	0.9667	1.0241	0.79	1.40	0.585
	2.00	27	1.0096	0.14103	0.02714	0.9538	1.0654	0.75	1.32	
	3.00	18	1.0294	0.14210	0.03349	0.9588	1.1001	0.76	1.39	
	Total	108	1.0046	0.12547	0.01207	0.9807	1.0286	0.75	1.40	
AscA/AA	1.00	63	1.1933	0.17621	0.02220	1.1490	1.2377	0.79	1.65	0.486
	2.00	27	1.2448	0.24061	0.04631	1.1496	1.3400	0.87	2.00	
	3.00	18	1.2017	0.12045	0.02839	1.1418	1.2616	0.94	1.45	
	Total	108	1.2076	0.18665	0.01796	1.1720	1.2432	0.79	2.00	

***AA*** ventriculo-aortic junction**,** ***AscA*** ascending aorta**,** ***SV*** sinus Valsalva**,** ***SVI*** sinus Valsalva index, ***STJ*** sinotubular junction, ***STJI*** sinotubular junction index

Additional testing with Spearman Correlation Coefficient test confirmed the ANOVA results. Statistically significant and positive correlations were established between grades and parameters STJ and AscA. Testing also revealed significant positive association of echocardiographic parameter STJI and the defined grades (Table 4).

**Table 4:** Spearman Correlation Coefficient for correlation of grades and echocardiographic parameters

	AA	SV	STJ	AscA	SVI	STJI	STJAA	AscAA
Grade	0.189	0.132	0.268^**^	0.250^**^	0.095	0.203^*^	0.085	0.076

*p < 0.05; **p < 0.01

*AA* ventriculo-aortic junction, *AscA* ascending aorta, *SV* sinus Valsalva, *SVI* sinus Valsalva index, *STJ* sinotubular junction, *STJI* sinotubular junction index

### Influence of aging

Patients age did not differ significantly among different grades as confirmed with ANOVA and post hoc Tukey HSD analysis (F = 0.398; *p* = 0.673 (*p* > 0.05)). Also, when distribution of grades was counted in a group <65 years and ≥65 years, Pearson Chi-square test revealed no statistically significant difference (Pearson Chi-square = 0.405; *p* = 0.817 (p > 0.05)).

**Fig. 3:**
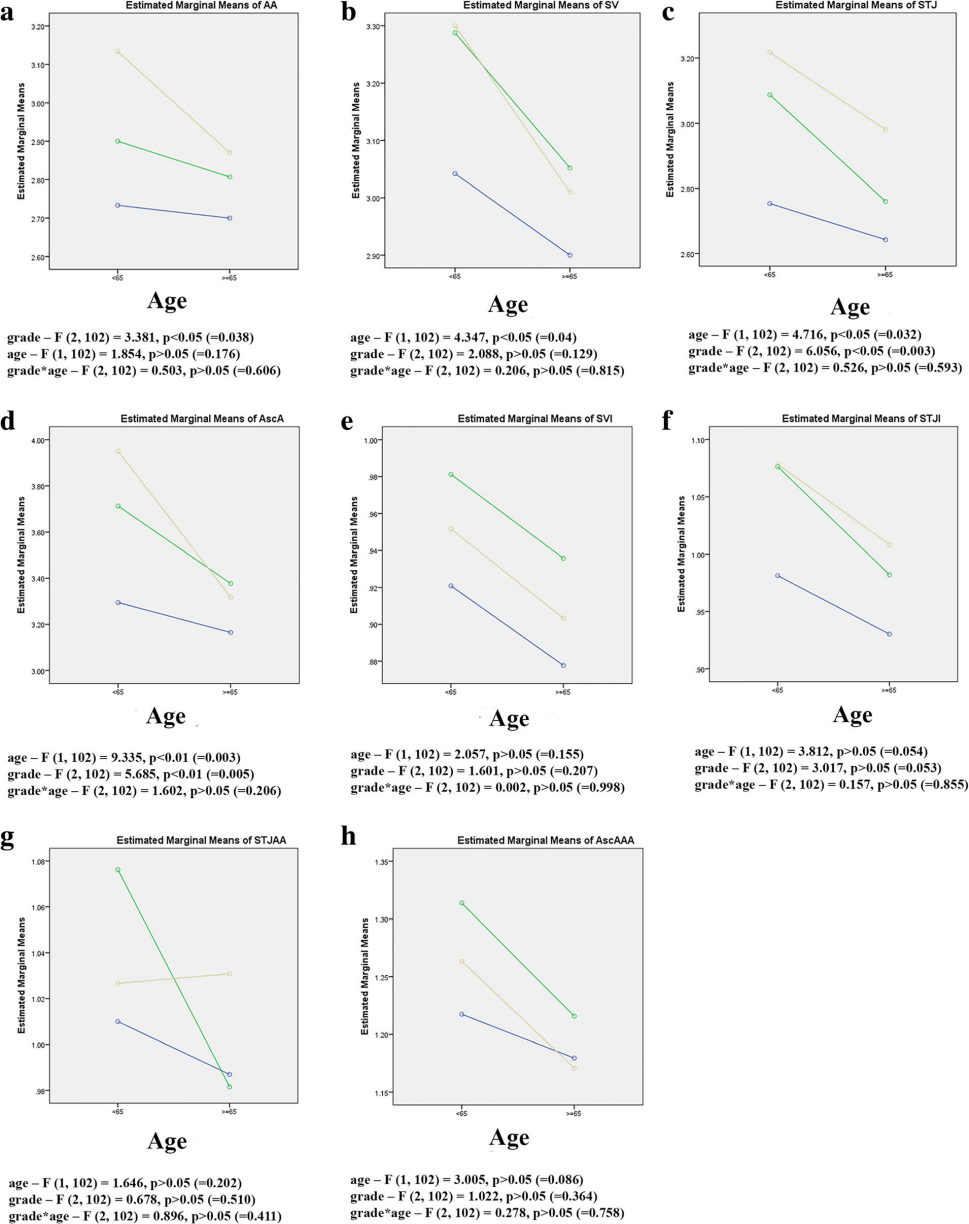
Influence of age and grade to echocardiographic parameters – there is an increase in values of echocardiographic parameters with grade and a decrease with age. However differences are not statistically significant (blue - grade 1; green – grade 2; beige – grade 3): simultaneous effects of age and grade to **a**) diameters at the level of ventriculo-aortic junction (AA), **b**) diameters at the level of sinus Valsalva (SV), **c**) diameters at the level of sinotubular junction (STJ), **d**) the largest diameter of visualized ascending aorta (AscA), **e**) values of sinus Valsalva index (SVI), **f**) values of sinotubular junction index (STJI), **g**) STJ/AA and **h**) AscA/AA

Two-way ANOVA that tested influence of age and grade to different echocardiographic parameters revealed no statistically significant difference (Fig. 3).

### Influence of gender

**Fig. 4:**
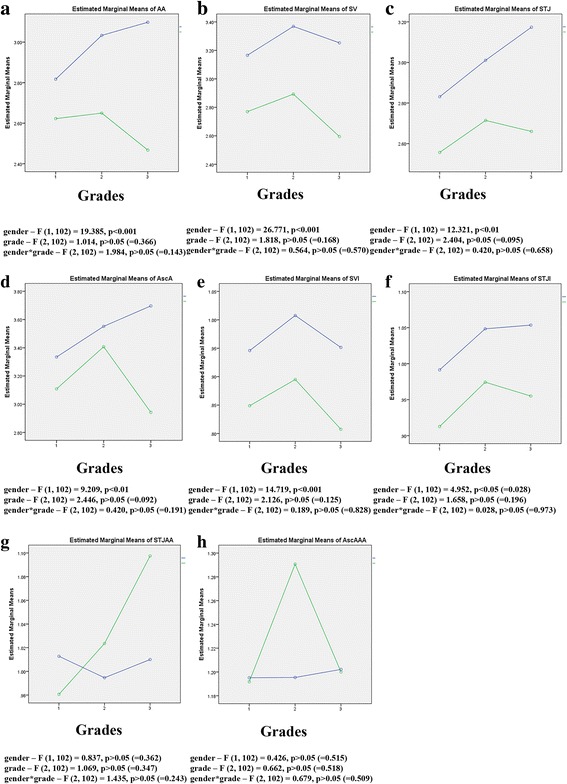
Influence of gender and grade to echocardiographic parameters: simultaneous effects of gender and grade to **a**) diameters at the level of ventriculo-aortic junction (AA), **b)** diameters at the level of sinus Valsalva (SV), **c**) diameters at the level of sinotubular junction (STJ), **d**) the largest diameter of visualized ascending aorta (AscA), **e**) values of sinus Valsalva index (SVI), **f**) values of sinotubular junction index (STJI), **g**) STJ/AA and **h**) AscA/AA

Distribution of grades did not differ among genders as confirmed with Pearson Chi-Square test (5.849; *p* = 0.054 (p > 0.05)). All echocardiographic parameters except indexes STJ/AA and AscA/AA were statistically significantly higher in males than in females, but simultaneous effects of grade and gender to echocardiographic parameters was not statistically significant as confirmed with Two-Way ANOVA (Fig. 4).

### Multifactorial analysis

Echocardiographic parameters had no differences when tested for the presence or absence of arterial hypertension, diabetes mellitus and atherosclerosis (data not shown).

Application of Regression Analysis by using multiple predictors: age, gender, presence of arterial hypertension, diabetes mellitus, atherosclerosis with changes of grade identified statistically significant differences among echocardiographic parameters (Table 5).

**Table 5:** Regression Analysis predicting significance of multiple variables and grades to values of echocardiographic parameters

Predictors	Dependant variable											
	AA			SV			STJ			AscA		
	B	95% C.I.		B	95% C.I.		B	95% C.I.		B	95% C.I.	
HTA (arterial hypertension)	0.089	−0.078	0.256	0.030	−0.151	0.211	0.079	−0.105	0.263	0.162	−0.058	0.382
DM (diabetes mellitus)	−0.051	−0.226	0.124	−0.009	−0.198	0.181	0.017	−0.176	0.210	−0.186	−0.416	0.045
Atherosclerosis	0.092	−0.042	0.226	0.028	−0.117	0.173	0.030	−0.117	0.178	0.070	−0.106	0.246
Grade	0.080	−0.018	0.178	0.036	−0.070	0.142	0.148	0.039	0.256	0.134	0.005	0.263
Gender	−0.289	−0.440	−0.139	−0.428	−0.591	−0.265	−0.298	−0.464	−0.132	−0.265	−0.463	−0.067
Age	−0.003	−0.012	0.006	−0.006	−0.016	0.003	−0.009	−0.019	0.001	−0.013	−0.025	−0.002
F	4.249			5.705			5.107			3.995		
p	0.001			<0.001			<0.001			0.001		
Predictors	Dependant variable											
	SVI			STJI			STJ/AA			AscA/AA		
	B	95% C.I.		B	95% C.I.		B	95% C.I.		B	95% C.I.	
HTA (arterial hypertension)	0.024	−0.032	0.024	0.035	−0.033	0.102	−0.016	−0.071	0.040	0.162	−0.058	0.382
DM (diabetes mellitus)	−0.002	−0.061	−0.002	0.005	−0.066	0.076	0.028	−0.030	0.086	−0.186	−0.416	0.045
Atherosclerosis	0.013	0.032	0.013	0.008	−0.046	0.061	−0.030	−0.074	0.015	0.070	−0.106	0.246
Grade	0.007	−0.026	0.007	0.036	−0.004	0.075	0.019	−0.014	0.051	0.134	0.005	0.263
Gender	−0.100	−0.151	−0.100	−0.075	−0.135	−0.014	0.000	−0.050	0.050	−0.265	−0.463	−0.067
Age	−0.001	−0.004	−0.001	−0.003	−0.007	0.000	0.002	−0.005	0.001	−0.013	−0.025	−0.002
F	3.268			2.701			1.055			0.800		
p	0.006			0.018			0.395			0.572		

**Fig. 5:**
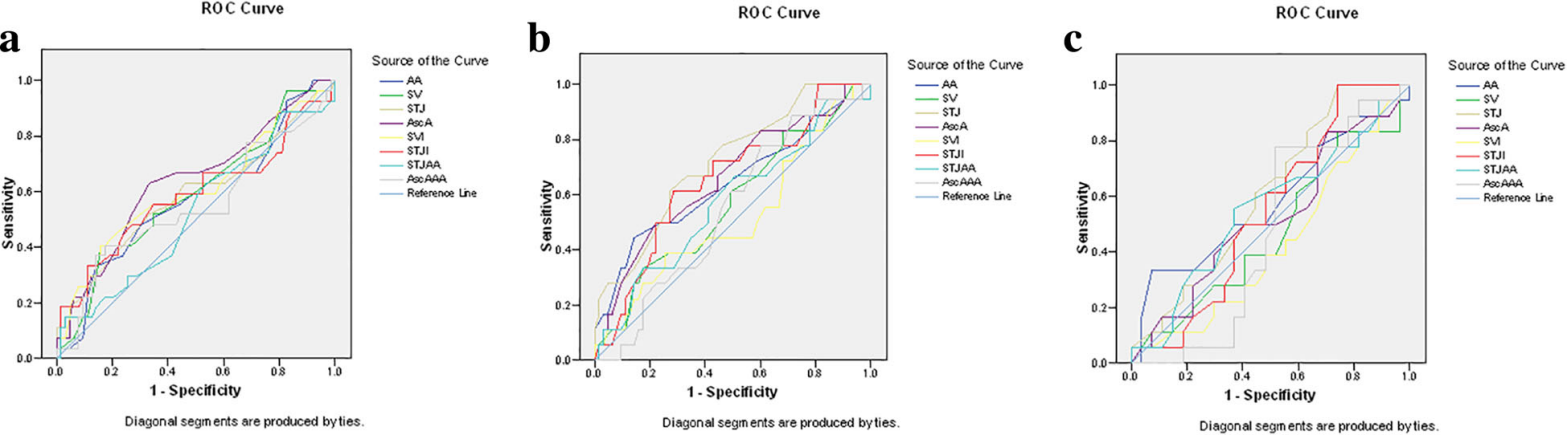
ROC Curves test the sensitivity and specificity of different echocardiographic parameters for different grades for parameters AscA (**a**), STJ (**b**) and STJI (**c**)

Based on the regression analysis ROC Curves were constructed to test the sensitivity and specificity of different echocardiographic parameters for different grades (Fig. 5).

According to ROC Curves analysis following threshold values were detected to identified specific grades (Table 6).

**Table 6:** Threshold values of echocardiographic parameters that identify different grades

Variable Classification variable	Criterion	Sensitivity	95% CI	Specificity	95% CI	+LR	95% CI	-LR	95% CI
AscA Grade 2 vs. 1	>3.3 *	62.96	42.4–80.6	66.67	53.7–78.0	1.89	1.3–2.6	0.56	0.3–1.0
AscA Grade 3 vs 1	>3.5 *	50.00	26.0–74.0	77.78	65.5–87.3	2.25	1.4–3.6	0.64	0.3–1.2
STJ Grade 3 vs. 1	>2.9 *	61.11	35.7–82.7	73.02	60.3–83.4	2.26	1.5–3.4	0.53	0.3–1.1
STJI Grade 3 vs 1	>1 *	61.11	35.7–82.7	71.43	58.7–82.1	2.14	1.4–3.2	0.54	0.3–1.1

*statistically significant

*AscA* ascending aorta, *STJ* sinotubular junction, *STJI* sinotubular junction index

## Discussion

The debate about concomitant replacement of ascending aorta with aortic valve replacement spins around aortic diameter, etiology of valve disease, structural changes in the aortic wall and the influence of mechanical stress on the aortic wall. Our study focuses on TAV stenosis and structural derangements in the aortic wall that it causes.

According to the current guidelines, surgery should be considered in patients who have aortic root disease (whatever the severity of aortic regurgitation or stenosis) with maximal ascending aortic diameter ≥ 45 mm for patients with Marfan syndrome with risk factors, ≥50 mm for patients with bicuspid valve with risk factors, and ≥55 mm for other patients [13].

Aortic valve disease is associated with the ascending aortic dilatation because of the “hemodynamic burdens caused by forceful jets” [14, 15]. Due to mechanical stress, the size of the dilatation is related to the degree of turbulence induced by the stenotic valve and the severity of stenosis [16]. However, it seems that there is no independent association between the severity of aortic stenosis and the aortic diameter, indicating that factors other than the aortic stenosis itself (geometry of aortic orifice, flow distribution pattern and histopathological changes in the aortic wall) could affect the echocardiographic parameters of the aorta [17].

Gaudino et al. [16] published the results of follow-up study of patients submitted to AVR only and showed moderate dilatation of the ascending aorta with the expansion rate of 0.3 ± 0.2 mm/year after 10 years postoperatively [16]. Similarly, Yasuda et al. [18] reported a mean ascending aorta expansion rate of 0.08 mm/m^2^/year in a series of 14 patients followed for 9.7 years after surgery [18]. They speculated that correction of the aortic stenosis in these patients stabilized the hemodynamics and prevented further development of the aortic wall changes. Andrus et al. [19] reported results of a vast study that comprised 107 patients with an aortic diameter ≥ 3.5 cm. He found no evidence of further dilation in the first 3 years after isolated AVR, and concluded that in patients with aortic valve stenosis and with accompanying mild or moderate ascending aortic dilatation (3.5 cm to 4.9 cm) AVR alone may be reasonable [19].

Botzenhardt et al. [20] have even described a reduction of the aortic diameter in 10 patients with pre-operative aortic diameter ≥ 4 cm, 4.8 years after the isolated valve surgery [20].

As opposite to these studies, Matsuyama et al. [21] concluded that the clinical course of patients with a dilated ascending aorta is unpredictable and that aortic events may occur in patients with an aortic diameter of <5 cm. The author also found that patients with TAV stenosis and a slightly dilated aorta are at risk of late aortic events. Therefore, suggested preventive aortic surgery and AVR, even in patients with slightly dilated ascending aorta with a diameter of 4 cm to 5 cm, except in cases of high operative risk [21].

Ergin et al. [22] advocate more liberal ascending aorta replacement in conjunction with AVR since it significantly improves postoperative outcome in comparison to patients with AVR and already dilated aorta [22].

Only few studies investigated histopathological defects of aortic wall elastic skeleton in patients with the aortic valve dysfunction, utilizing limited number of elastic skeleton parameters. Roberts et al. [11] using a semi-quantitative method, found that there is no significant loss of elastic fibers in patients with stenosis of the TAV as compared to the control group [11]. Bauer et al. [4] showed that the thickness of elastic lamellae is decreased and the distance between elastic lamellae is increased significantly in patients with dilatation of the ascending aorta and with TAV stenosis [4]. Bechtel et al. [23] found that patients with TAV stenosis and the ascending aorta dilatation have more severe defects of the ascending aorta than patients with bicuspid valve and the same degree of dilatation [23].

Von Kodolitsch et al. [24] concluded that any patient at aortic valve replacement with an aortic diameter ≥ 43 mm and the presence of aortic wall fragility, aortic thinning or aortic regurgitation, will likely benefit from prophylactic aortic surgery. The combined presence of these parameters identifies a disease process of the entire aortic root rather than isolated valve disease [24].

Tsutsumi et al. [25] portrayed clinical entity of the patients prone to postsurgical aortic complications. They suggested that patients with aortic regurgitation combined with systemic hypertension, male sex, and thinned or fragile aortic walls in patients with ascending aortic dilatation (≥45 mm diameter) at the time of aortic valve replacement, should be considered for concomitant replacement of the ascending aorta [25].

Beller et al. [26] found that, in cases of aortic stenosis, restored aortic valve competence (by replacing the diseased valve) is associated with increased aortic root motion, theoretically heightening the threat of dissection posed to the aortic wall by mechanical stress. Mechanical principles command to include the higher magnitude of aortic root motion during follow-up of patients after AVR as an additional risk factor for dissection [26].

We have previously found significant thinning of the ascending aorta wall and all its tunics in patients with aortic stenosis [27]. Similar changes have already been described in a different model of exaggerated hemodynamic forces and its influence to the arterial wall [28]. Rabkin, Jue and Tsang [29] proved echocardiographically that after the adjustment for body surface area, wall thickness of the sinus Valsalva is a good indicator of the aortic wall stress associated with the aortic valve sclerosis even in those cases when luminal diameters of the aorta are not dilated [29].

We applied Schlatmann and Becker grading system to demonstrate three different histopathological grades. Furthermore, our supposition is that these three grades follow the natural progression and evolution of aortic stenosis and its hemodynamic impact to the aortic wall. In our previous study, we confirmed significant progress of elastic lamellae disruption through different grades as well as spatial distribution of these changes in the aortic wall as they affect the internal media first [27]. These observations are in keeping with previous similar studies [6–8, 30, 31].

Grade 3 with destructive changes in numerous elastic lamellae and disorganization of smooth muscles resembled irreversible changes in the aortic wall.

Morphological and morphometric characteristics of elastic skeleton are changing during aging. Even the “perfect” internal thoracic artery is prone to elastic skeleton changes induced by aging [6, 7]. Nakashima et al. [30] proved that the number of elastic lamellae fenestrations increase with aging, as does the number of interlamellar elastic fibers, their ramifications and the number of their fenestrations [30]. It was very important to prove that the observed grades are not merely effects of aging. Our study showed there is no difference between patient age distribution in different histological grades. Dividing patients in two age groups (˂65 and ≥65), there is no difference in the distribution of grades. Described changes persisted in both groups of patients, younger and older than 65 years, they are potentiated with aging, but they are not the effect of aging entirely.

Combined effect of gender and grade had no effect to echocardiographic parameters and the distribution of grades among genders did not differ significantly.

Girdauskas et al. [32], using cardiac magnetic resonance imaging, showed that systolic transvalvular flow jet is hitting the right-lateral segment of the tubular ascending aorta, in patients with aortic valve stenosis. This finding confermed that we sampled aorta from the right place [32]. By using multiple predictors in the setting of Regression analysis, statistically significant differences among grades were reached for AA, SV, STJ, AscA and SVI. With further ROC curves analysis, threshold values for different grades were recognized. Grade 2 is identified in patients with AscA > 3.3 cm, while grade 3 is identified in patients with values of AscA > 3.5 cm, STJ > 2.9 cm and STJI > 1.

### Limitations of the study

Our study obviously lacks *post festum* echo analisys – to determine what happens with aortas in patients with different grades, following AVR. Nevertheless, we focused on proving the existence of different, progressive, histological changes in the aortic wall, and to correlate them with various echo parameters, in patients exclusively with severe stenosis of tricuspid aortic valve.

## Conclusion

Our findings strongly support the view that aortas of patients with TAV stenosis are submitted to hemodynamic stress that subsequently leads to gradual elastic lamellae disruption that could be histologically identified and graded. The changes in the aortic wall correlated statistically significant with echocardiographic parameters. Grade 2 is identified in patients with AscA > 3.3 cm, while grade 3 is identified in patients with values of AscA > 3.5 cm, STJ > 2.9 cm and STJI > 1. Although current guidelines suggest simultaneous replacement of ascending aorta with AVR when aortic diameter is ≥55 mm, we propose more radical approach, with diameter > 3.5 cm as a cutoff, in patients with severe TAV stenosis, especially in patients with long life expectancy.

## Abbreviations

AA: Ventriculo-aortic junction
AscA: Ascending aorta
AVR: Aortic valve replacement
BSA: Body surface area
GR: Grade
pSTJ: Predicted diameter at the level of sinotubular junction
pSV: Predicted diameter at the level of the sinus of Valsalva
STJ: Sinotubular junction
STJI: Sinotubular junction index
SV: Sinus Valsalva
SVI: Sinus Valsalva index
TAV: Tricuspid aortic valve
